# Fixed points of two interpolative cyclic contractions in *b*-metric spaces

**DOI:** 10.1016/j.heliyon.2025.e41667

**Published:** 2025-01-07

**Authors:** Darsana Devi, Pradip Debnath

**Affiliations:** Department of Mathematical Sciences, Tezpur University, Napaam, Assam - 784028, India

**Keywords:** 47H10, 54H25, 54E50, Fixed point, Contraction map, *b*-metric space, Complete metric space, Kannan type contraction

## Abstract

The *b*-metric space happens to be one of the of most significant and non-trivial generalizations of metric spaces. In this paper, we introduce the concepts of Kannan type and Ćirić-Reich-Rus type cyclic contractions in *b*-metric spaces via interpolation. Existence and uniqueness of fixed points of these two newly introduced contraction mappings have been studied and validated with suitable examples. Our paper also generalizes, extends and provides improvements to the results in the recent paper by Edraoui et al. (2023) [11].

## 1 Introduction and preliminaries

In 1922, Bakhtin [3] introduced a new direction in the structure of metric space by generalizing the triangular inequality and this concept was formally defined as *b*-metric space by Czerwik [6]. This implication of weakening the triangular inequality in the extended version of metric space makes this ambient space useful to measure ice floes [4], pattern matching [12] etc.

Definition 1.1 [3] Let B be a nonempty set and s≥1 be a given real number. A function b:B×B→[0,∞) is said to be a *b*-metric if for u,v,z∈B the following conditions are satisfied:

(a) b(u,v)=0⇔u=v,
(b) b(u,v)=b(v,u),
(c) b(u,v)≤s[b(u,z)+b(z,v)] (*b*-triangular inequality).

(B,b) is called a *b*-metric space (with constant *s*).

Note that every metric space is a *b*-metric space with s=1. However, converse of the above statement is not necessarily true. Let us consider the following example:

Let B=N. Define b:B×B→[0,∞) by

$$b(u,v)=\left\{ \begin{array}{c} 0,\text{if}\;u=v,\\ 4\alpha ,\text{if}\;u,v\in \{1,2\},\\ \alpha ,\text{if}\;u\;\text{or}\;v\notin \{1,2\}\;\text{and}\;u\neq v, \end{array}\right.$$

where α>0 is a constant.

Here (B,b) is a *b*-metric space with s=3. Now, b(1,2)=4α, b(1,3)=α, b(3,2)=α and b(1,3)+b(3,2)=2α.

Therefore, (B,b) is not a metric space as b(1,2)>b(1,3)+b(3,2).

Definition 1.2 [23] Let $\left(B,b_{1}\right)$ and $\left(C,b_{2}\right)$ be two *b*-metric spaces. A map I:B→C is said to be continuous at z∈B if $\lim_{n\to \infty }b_{2}(Iz_{n},Iz)=0$ for all $\{z_{n}\}\subset B$ with $\lim_{n\to \infty }b_{1}(z_{n},z)=0$.

I is continuous on B if and only if I is continuous at every point of B.

One of the basic topological property continuity plays a crucial role in metric fixed point theory. However, it is worth mentioning that *b*-metric space fails to be continuous as a function in general where as a metric is always continuous. So, the continuity can be taken as one of the main difference between a metric and a *b*-metric [23].

Definition 1.3 [14] Let (B,b) be a *b*-metric space. A sequence $\left\{u_{n}\right\}$ in B is said to be

(a) Cauchy if and only if $b(u_{n},u_{m})\to 0$ as n,m→∞.
(b) Convergent if and only if there exists u∈B such that $b(u_{n},u)\to 0$ as n→∞ and we write $\lim_{n⟶\infty }u_{n}=u$.
(c) The *b*-metric space (B,b) is complete if every Cauchy sequence is convergent.

The extension of Banach fixed point result to the class of cyclic map and the concept of cyclical contractive mapping was introduced by Kirk et al. [22]. They proved the existence of unique fixed point in different types of cyclical contractive mappings in a complete metric space.

Definition 1.4 [22] Let (B,b) be a metric space. Let P and Q be two nonempty subsets of B. A mapping I:P∪Q→P∪Q is said to be a cyclic mapping provided,

I(P)⊆Q,I(Q)⊆P.

Kirk et al. proved the following Fixed point theorem for cyclical contraction map:

Theorem 1.5 *[22]* *Let* (B,b) *a complete metric space and let* P *and* Q *be two nonempty subsets of* B *. Suppose that* I:P∪Q→P∪Q *is a cyclic contraction and there exists* k∈(0,1) *such that* b(Iu,Iv)≤kb(u,v) *for all* u∈P *and* v∈Q *. Then,* I *has a unique fixed point in* P∩Q *.*

Karapinar [17] introduced the concept of interpolation in Kannan [16] type contractions and proved the existence and uniqueness of fixed point for such contractions. Similarly, the concept of interpolation was applied for Reich type contractions in [18] and Hardy-Rogers type contractions in [19]. For more such relevant recent literature, we refer to [1], [2], [5], [20], [21].

Definition 1.6 [16], [17], [18] Let (B,b) be a metric space and let P and Q be two nonempty subsets of B.

A cyclic map I:P∪Q→P∪Q is said to be a:

(a) Kannan type cyclic contraction if there exists $k\in (0,\frac{1}{2})$ such that
  b(Iu,Iv)≤k[b(Iu,u)+b(Iv,v)],∀u∈P,∀v∈Q.
(b) Reich type cyclic contraction if there exists $k\in (0,\frac{1}{3})$ such that
  b(Iu,Iv)≤k[b(u,v)+b(Iu,u)+(Iv,v)],∀u∈P,∀v∈Q.

Researchers have extended the Kannan's and Reich's fixed point theorems in several ways and many interesting results are established. To delve into their work and the future outcomes we refer to the works of Aydi et al. [24], Debnath et al. [7], [8], [9], [10], Górnicki [13], Karapinar et al. [17], [19], Lu et al. [23].

In this paper, we use the results of Kannan and Reich [15], [16], [25], [26] and introduce their interpolative versions for cyclic contraction in a complete *b*-metric space.

**Improvements to the results in a recent paper:**

As discussed in the abstract, our paper also generalizes, extends and provides improvements to the results in the recent paper by Edraoui et al. [Appl. Gen. Topol. 24 (2) 2023, 247-252] [11].

For clarity of expression and comparison, we state below the two main results of [11], which will facilitate readers to compare the results at a glance and more clearly identify the improvements in the results of this paper.

Theorem 2.2 *[11]* *Let* (B,b) *be a complete metric space and let* P *and* Q *be two non-empty subsets of* B *. If* I:P∪Q→P∪Q *is an interpolative Kannan-type cyclic contraction, then* I *has a unique fixed point in* P∩Q *.*

Theorem 2.4 *[11]* *Let* (B,b) *be a complete metric space and let* P *and* Q *be nonempty subsets of* B *. Let* I:P∪Q→P∪Q *be an interpolative Ćirić-Reich-Rus-type cyclic contraction. Then* I *has a unique fixed point in* P∩Q *.*

In that paper, in the proof Theorem 2.2 and Theorem 2.4, the authors have concluded that in a complete metric space (B,b), if $\sum_{n=1}^{\infty }b(u_{n+1},u_{n})<\infty$, then the sequence $\left\{u_{n}\right\}$ is Cauchy.

In fact, from the condition $\sum_{n=1}^{\infty }b(u_{n+1},u_{n})<\infty$, if one concludes that

$$\lim_{n\to \infty }b(u_{n+1},u_{n})=0,$$

then this does not guarantee that $\left\{u_{n}\right\}$ is Cauchy. Indeed, if we consider R with usual metric b(u,v)=|u−v| and the sequence $u_{n}=1+\frac{1}{2}+\cdots +\frac{1}{n}$, then $b(u_{n+1},u_{n})=\frac{1}{n+1}\to 0$ as n→∞.

But, $b(u_{2n},u_{n})=\frac{1}{n+1}+\frac{1}{n+2}+\cdots +\frac{1}{2n}\to \ln2$ as n→∞.

In the current paper, we have adopted a new technique of proof to avoid any ambiguity or confusion. Moreover, our results have been established in the new setting of a *b*-metric space.

First, we introduce a proper generalization of Kannan's fixed point theorem for cyclic contraction and further introduce a Ćirić-Reich-Rus type generalization of the same.

## 2 Interpolative Kannan type cyclic contraction

Definition 2.1 Let (B,b) be a complete *b*-metric space and let P and Q be two non-empty subsets of B. A cyclic map I:P∪Q→P∪Q is said to be an interpolative Kannan-type cyclic contraction if there exist k∈[0,1) and α∈[0,1) such that

(2.1) $$b(Iu,Iv)\leq k{\left[b(Iu,u)\right]}^{\alpha }{\left[b(Iv,v)\right]}^{1-\alpha }$$

for all (u,v)∈P×Q with u,v∉Fix(I), where Fix(I) denotes the set of all fixed points of I.

Theorem 2.2 *Let* (B,b) *be a complete b-metric space and let* P *and* Q *be two non-empty subsets of* B *. If* I:P∪Q→P∪Q *is an interpolative Kannan-type cyclic contraction, then* I *has a unique fixed point in* P∩Q *.*

Proof Let (B,b) be a complete *b*-metric space and I:P∪Q→P∪Q be an interpolative Kannan type cyclic contraction. Let us define a sequence $\left\{u_{n}\right\}$ in (B,b) such that

(2.2) $$I(u_{n})=u_{n+1},\text{ for all}\;n\in N\cup \{0\}.$$

Fix $u_{0}\in P$ then $I(u_{0})\in Q$, $I^{2}(u_{0})\in P$, $I^{3}(u_{0})\in Q$ and so on. By using equation (2.2) we can write,

$$u_{1}=I(u_{0})u_{2}=I^{2}(u_{0})u_{3}=I^{3}(u_{0})$$

and so on. In general, $u_{n}=I^{n}(u_{0})$ for all n∈N∪{0}.

Since I:P∪Q→P∪Q is an interpolative cyclic contraction in (B,b), therefore the equation 2.1 becomes

(2.3) $$b(I^{2}(u_{0}),I(u_{0}))\leq k{\left[b(I^{2}(u_{0}),I(u_{0}))\right]}^{\alpha }{\left[b(I(u_{0}),u_{0})\right]}^{1-\alpha }\Rightarrow {\left[b(I^{2}(u_{0}),I(u_{0}))\right]}^{1-\alpha }\leq k{\left[b(I(u_{0}),u_{0})\right]}^{1-\alpha }\Rightarrow [b(I^{2}(u_{0}),I(u_{0}))]\leq k^{\frac{1}{1-\alpha }}[b(I(u_{0}),u_{0})]\Rightarrow b(I^{2}(u_{0}),I(u_{0}))\leq tb(I(u_{0}),u_{0}),\text{ where }t=k^{\frac{1}{1-\alpha }}\in [0,1)\Rightarrow b(u_{2},u_{1})\leq tb(u_{1},u_{0}).$$

In general, using inequality (2.3), we can write

$$b(u_{n+1},u_{n})\leq t^{n}b(u_{1},u_{0})\text{ for all }n\in N\cup \{0\}.$$

This implies that $\sum_{n=1}^{\infty }b(u_{n+1},u_{n})\leq \sum_{n=1}^{\infty }t^{n}b(u_{1},u_{0})$ for all n∈N∪{0}.

As $\sum_{n=1}^{\infty }t^{n}b(u_{1},u_{0})$ is a convergent series (being geometric), therefore by comparison test the series $\sum_{n=1}^{\infty }b(u_{n+1},u_{n})$ is also converges.

Therefore $b(u_{n+1},u_{n})\to 0$ as n→∞.

Now, for all m∈N with m>n and using (2.1), we have

(2.4) $$b(u_{n},u_{m})=b(I^{n}(u_{0}),I^{m}(u_{0}))\leq k{\left[b(I^{n}(u_{0}),I^{n-1}(u_{0}))\right]}^{\alpha }{\left[b(I^{m}(u_{0}),I^{m-1}(u_{0}))\right]}^{1-\alpha }\leq k{\left[b(u_{n},u_{n-1})\right]}^{\alpha }{\left[b(u_{m},u_{m-1})\right]}^{1-\alpha }.$$

Taking limit in (2.4) as n→∞, we get $b(u_{n},u_{n-1})\to 0$ and $b(u_{m},u_{m-1})\to 0$.

Therefore $b(u_{n},u_{m})\to 0$ as n→∞.

Hence $\left\{u_{n}\right\}$ is a Cauchy sequence in B. (B,b) being a complete metric space, every Cauchy sequence of B converges in B.

We can write, $b(u_{n},\theta )\to 0$ as n→∞ for some θ∈B.

Notice that $\left\{u_{2n}\right\}$ is a sequence in P and $\left\{u_{2n+1}\right\}$ is a sequence in Q having the same limit *θ* [19]. Therefore

θ∈P∩Q.

Now, we have to show that *θ* is a fixed point of I:P∪Q→P∪Q.

Since (B,b) is a *b*-metric space, therefore for s≥1 we can write

(2.5) $$b(\theta ,I\theta )\leq s[b(\theta ,u_{n+1})+b(u_{n+1},I\theta )]\leq s[b(\theta ,u_{n+1})+b(I(u_{n}),I\theta )]\leq sb(\theta ,u_{n+1})+sb(I(u_{n}),I\theta )\leq sb(\theta ,u_{n+1})+s.k{\left[b(I(u_{n}),u_{n})\right]}^{\alpha }{\left[b(I\theta ,\theta )\right]}^{1-\alpha }\leq sb(\theta ,u_{n+1})+s.k{\left[b(u_{n+1},u_{n})\right]}^{\alpha }{\left[b(I\theta ,\theta )\right]}^{1-\alpha }.$$

Taking limit in (2.5) as n→∞, we have $b(\theta ,u_{n+1})\to 0$ and $b(u_{n},u_{n+1})\to 0$. This implies b(θ,Iθ)=0 and hence Iθ=θ.

Now, let w∈P∩Q and I(w)=w such that w≠θ.

From equation (2.1), $b(w,\theta )=b(Iw,I\theta )\leq k{\left[b(Iw,w)\right]}^{\alpha }{\left[b(I\theta ,\theta )\right]}^{1-\alpha }$.

Hence b(w,θ)=0.

Therefore we get, w=θ. Thus, *θ* is the unique fixed point of I:P∪Q→P∪Q in the complete *b*-metric space (B,b). □

Example 2.3 Let B=[0,1] and $b(u,v)={\left(u-v\right)}^{2}$ for all u,v∈B. Then (B,b) is a complete *b*-metric space with s=2.

**Figure 1:**
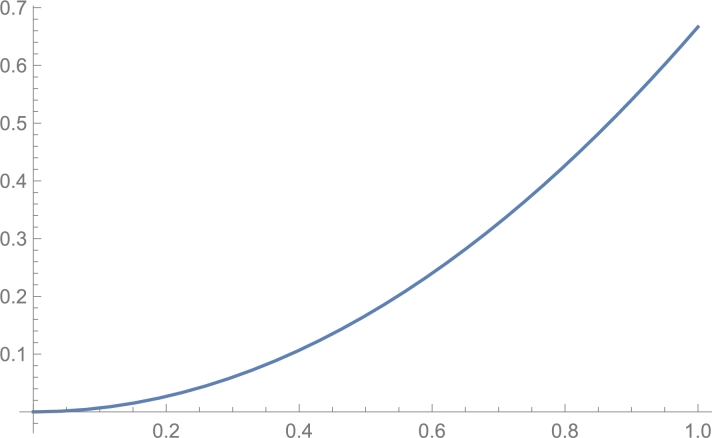
Plot of the function I:B→B.

Define the self-map (shown in Fig. 1) I:B→B by $Iu=\frac{2}{3}u^{2}$. If we consider u,v∈B∖Fix(I), then, clearly u,v∈(0,1]. Thus conditions of (2.1) are satisfied for $\alpha =\frac{1}{3}$.

Thus, I is an interpolative Kannan type cyclic contraction and 0 is the unique fixed point of I.

## 3 Interpolative Ćirić-Reich-Rus-type cyclic contraction

In this section, we introduce and study interpolative Ćirić-Reich-Rus-type cyclic contraction in a *b*-metric space.

Definition 3.1 Let (B,b) be a complete *b*-metric space. Let P and Q be two nonempty subsets of B. A cyclic map I:P∪Q→P∪Q is said to be an interpolative Ćirić-Reich-Rus-type cyclic contraction if there exist k∈[0,1) and positive reals α,β,α+β<1 such that

(3.1) $$b(Iu,Iv)\leq k{\left[b(u,v)\right]}^{\beta }{\left[b(Iu,u)\right]}^{\alpha }{\left[b(Iv,v)\right]}^{1-\alpha -\beta }$$

for all (u,v)∈P×Q withu,v∉Fix(I).

Theorem 3.2 *Let* (B,b) *be a complete b-metric space and let* P *and* Q *be nonempty subsets of* B *. Let* I:P∪Q→P∪Q *be an interpolative Ćirić-Reich-Rus-type cyclic contraction. Then* I *has a unique fixed point in* P∩Q *.*

Proof Let (B,b) be a complete *b*-metric space and I:P∪Q→P∪Q be an interpolative Ćirić-Reich-Rus - type cyclic contraction. Let us define a sequence $\left\{u_{n}\right\}$ in B such that

$$I(u_{n})=u_{n+1},\text{ for all}\;n\in N\cup \{0\}.$$

Fix $u_{0}\in P$ then $I(u_{0})\in Q,\;I^{2}(u_{0})\in P,\;I^{3}(u_{0})\in Q$ and so on. Therefore we can write,

$$u_{1}=I(u_{0})u_{2}=I^{2}(u_{0})u_{3}=I^{3}(u_{0})$$

and so on. In general $u_{n}=I^{n}(u_{0})$ for all n∈N∪{0}.

Since I:P∪Q→P∪Q is an interpolative cyclic contraction in (B,b), therefore the equation (3.1) becomes

(3.2) $$b(I^{2}(u_{0}),I(u_{0}))\leq k{\left[b(I^{2}(u_{0}),I(u_{0}))\right]}^{\beta }{\left[b(I^{2}(u_{0}),I(u_{0}))\right]}^{\alpha }{\left[b(I(u_{0}),u_{0})\right]}^{1-\alpha -\beta }\Rightarrow [b(I^{2}(u_{0}),I(u_{0}))]\leq k{\left[b(I(u_{0}),I(u_{0}))\right]}^{\alpha +\beta }{\left[b(I(u_{0}),u_{0})\right]}^{1-\alpha -\beta }\Rightarrow {\left[b(I^{2}(u_{0}),I(u_{0}))\right]}^{1-\alpha -\beta }\leq k{\left[b(I^{2}(u_{0}),u_{0})\right]}^{1-\alpha -\beta }\Rightarrow [b(I^{2}(u_{0}),I(u_{0}))]\leq k^{\frac{1}{1-\alpha -\beta }}[b(I(u_{0}),u_{0})]\Rightarrow b(I^{2}(u_{0}),I(u_{0}))\leq tb(I(u_{0}),u_{0});\text{ where }t=k^{\frac{1}{1-\alpha -\beta }}\in [0,1)\Rightarrow b(u_{2},u_{1})\leq tb(u_{1},u_{0}).$$

In general, using (3.2), we can write

$$b(u_{n+1},u_{n})\leq t^{n}b(u_{1},u_{0})\text{ for all}\;n\in N\cup \{0\}.$$

This implies that

$$\sum_{n=1}^{\infty }b(u_{n+1},u_{n})\leq \sum_{n=1}^{\infty }t^{n}b(u_{1},u_{0}).$$

Since $\sum_{n=1}^{\infty }t^{n}b(u_{1},u_{0})$ converges, therefore by comparison test $\sum_{n=1}^{\infty }b(u_{n+1},u_{n})$ is also convergent. Therefore $b(u_{n+1},u_{n})\to 0$ as n→∞.

Now, for all m∈N with m>n we have

(3.3) $$b(u_{n},u_{m})=b(I^{n}(u_{0}),I^{m}(u_{0}))\leq k{\left[b(I^{n-1}(u_{0}),I^{m-1}(u_{0}))\right]}^{\beta }{\left[b(I^{m}(u_{0}),I^{m-1}(u_{0}))\right]}^{\alpha }{\left[b(I^{n}(u_{0}),I^{n-1}(u_{0}))\right]}^{1-\alpha -\beta }\leq k{\left[b(u_{n-1},u_{m-1})\right]}^{\beta }{\left[b(u_{m},u_{m-1})\right]}^{\alpha }{\left[b(u_{n},u_{n-1})\right]}^{1-\alpha -\beta }.$$

Taking limit in (3.3) as n→∞ we get $b(u_{n},u_{n-1})\to 0$ and $b(u_{m},u_{m-1})\to 0$.

Therefore $b(u_{n},u_{m})\to 0$ as n→∞.

Hence $\left\{u_{n}\right\}$ is a Cauchy sequence in a *b*-metric space (B,b). Since (B,b) is a complete *b*-metric space so every Cauchy sequence converges in B.

We can write $b(u_{n},\theta )\to 0$ as n→∞ for some θinB.

Notice that $\left\{u_{2n}\right\}$ is a sequence in P and $\left\{u_{2n+1}\right\}$ is a sequence in Q having the same limit *θ*. Therefore

θ∈P∩Q.

Now, we have to show *θ* is the fixed point of I:P∪Q→P∪Q. Since (B,b) is a *b*-metric space, therefore for s≥1 we can write

(3.4) $$b(\theta ,I\theta )\leq s[b(\theta ,u_{n+1})+b(u_{n+1},I\theta )]\leq s[b(\theta ,u_{n+1})+b(I(u_{n}),I(\theta ))\leq sb(\theta ,u_{n+1})+sb(I(u_{n}),I\theta )\leq sb(\theta ,u_{n+1})+s.k{\left[b(u_{n},\theta )\right]}^{\beta }{\left[b(I(u_{n}),u_{n})\right]}^{\alpha }{\left[b(I\theta ,\theta )\right]}^{1-\alpha -\beta }\leq sb(\theta ,u_{n+1})+s.k{\left[b(u_{n},\theta )\right]}^{\beta }{\left[b(u_{n+1},u_{n})\right]}^{\alpha }{\left[b(I\theta ,\theta )\right]}^{1-\alpha -\beta }.$$

Taking limit in (3.4) as n→∞ we get $b(\theta ,u_{n+1})\to 0$ and $b(u_{n},u_{n+1})\to 0$.

This implies that b(θ,Iθ)=0 and hence Iθ=θ.

Now, let w∈P∩Q and I(w)=w such that w≠θ. Then from (3.1),

$$b(w,\theta )=b(Iw,I\theta )\leq k{\left[b(w,\theta )\right]}^{\beta }{\left[b(Iw,w)\right]}^{\alpha }{\left[b(I\theta ,\theta )\right]}^{1-\alpha -\beta }.$$

Hence b(w,θ)=0, i.e., w=θ.

Therefore, *θ* is the unique fixed point of I:P∪Q→P∪Q in the complete *b*-metric space (B,b). □

Example 3.3 Let B={u,v,z} and define b:B×B→[0,∞) as b(u,v)=0 if and only if u=v, b(u,v)=b(v,u) for all u,v∈M. Further, b(u,v)=1,b(u,z)=2.3,b(v,z)=1.1. Then (B,b) is a complete *b*-metric space with $s=\frac{23}{21}$, however it is not a metric space.

Define the self-map I:B→B by

$$Iu=\left\{ \begin{array}{c} u,\text{if}\;u=u\text{ or }u=v\\ v,\text{if}\;u=z. \end{array}\right.$$

Thus, we have

$$b(Iu,Iv)=\left\{ \begin{array}{c} b(u,u)=0,\text{if}\;u\neq z,v\neq z\\ b(v,u)=1,\text{if}\;u=z,v\neq z\\ b(u,v)=1,\text{if}\;u\neq z,v=z\\ b(v,v)=0,\text{if}\;u=z,v=z. \end{array}\right.$$

Now, if we consider u,v∈B∖Fix(I), then clearly the maximum value that b(Iu,Iv) can attain is 1. Thus, the inequality (3.1) and the conditions of Theorem 3.2 are satisfied for k=0.01, $\alpha =\frac{1}{2},\beta =\frac{1}{3}$. In this case, I has the unique fixed point u=0.

## 4 Conclusion

In conclusion, this paper significantly advances the study of *b*-metric spaces by introducing and exploring Kannan type and Ćirić-Reich-Rus type cyclic contractions. Through interpolation, we have established the existence and uniqueness of fixed points for these newly defined contraction mappings. Additionally, our findings extend and refine the results presented in the recent work by Edraoui et al., offering broader generalizations and corrections. We have adopted a new technique of proof to avoid error and ambiguity. These contributions not only enhance the theoretical framework of *b*-metric spaces but also provide practical examples that validate our results, thereby enriching the understanding of cyclic contractions in this context.

## Funding

This research received no external funding.

## CRediT authorship contribution statement

**Darsana Devi:** Writing – original draft, Validation, Investigation, Formal analysis. **Pradip Debnath:** Writing – review & editing, Visualization, Validation, Supervision, Software, Methodology, Investigation, Formal analysis, Conceptualization.

## Declaration of Competing Interest

The authors declare the following financial interests/personal relationships which may be considered as potential competing interests: Author Pradip Debnath is an Associate Editor of the journal Heliyon - Mathematics. If there are other authors, they declare that they have no known competing financial interests or personal relationships that could have appeared to influence the work reported in this paper.

## Data Availability

No new data was generated or used in this study.

## References

[1] Afshari H., Aydi H., Karapinar E. (2020). On generalized α−ψ- Geraghty contractions on b-metric spaces. Georgian Math. J..

[2] Alghamdi M.A., Gulyaz-Ozyurt S., Karapinar E. (2020). A note on extended Z-contraction. Mathematics.

[3] Bakhtin I.A. (1989). The contraction mapping in almost metric spaces. Funct. Anal. Gos. Ped. Inst. Unianowsk.

[4] Banfield J. (1991). Automated tracking of ice floes: a stochastic approach. IEEE Trans. Geosci. Remote Sens..

[5] Bilazeroglu S., Yalcin C. (2024). A fixed point theorem for Ćirić type contraction on interpolative metric spaces. J. Nonlinear Convex Anal..

[6] Czerwik S. (1993). Contraction mappings in *b*-metric spaces. Acta Math. Univ. Ostrav..

[7] Debnath P. (2022). Banach, Kannan, Chatterjea, and Reich–type contractive inequalities for multivalued mappings and their common fixed points. Math. Methods Appl. Sci..

[8] Debnath P. (2022). New common fixed point theorems for Górnicki-type mappings and enriched contractions. São Paulo J. Math. Sci..

[9] Debnath P., Mitrović Z., Radenović S. (2020). Interpolative Hardy-Rogers and Reich-Rus-Ćirić type contractions in *b*-metric spaces and rectangular *b*-metric spaces. Mat. Vesn..

[10] Debnath P., Srivastava H.M. (2020). New extensions of Kannan's and Reich's fixed point theorems for multivalued maps using Wardowski's technique with application to integral equations. Symmetry.

[11] Edraoui M., El Koufi A., Semami S. (2023). Fixed points results for various types of interpolative cyclic contraction. Appl. Gen. Topol..

[12] Fagin R., Stockmeyer L. (1998). Relaxing the triangle inequality in pattern matching. Int. J. Comput. Vis..

[13] Górnicki J. (2017). Fixed point theorems for Kannan type mappings. J. Fixed Point Theory Appl..

[14] Kadak U. (2014). On the classical sets of sequences with fuzzy b-metric. Gen. Math. Notes.

[15] Kannan R. (1968). Some results on fixed points. Bull. Calcutta Math. Soc..

[16] Kannan R. (1969). Some results on fixed points–II. Am. Math. Mon..

[17] Karapinar E. (2018). Revisiting the Kannan type contractions via interpolation. Adv. Theory Nonlinear Anal. Appl..

[18] Karapinar E., Agarwal R., Aydi H. (2018). Interpolative Reich–Rus–Ćirić type contractions on partial metric spaces. Mathematics.

[19] Karapinar E., Alqahtani O., Aydi H. (2018). On interpolative Hardy-Rogers type contractions. Symmetry.

[20] Karapinar E., Chifu C. (2020). Results in wt-distance over b-metric spaces. Mathematics.

[21] Karapinar E., Fulga A., Petrusel A. (2020). On Istratescu type contractions in b-metric spaces. Mathematics.

[22] Kirk W.A., Srinivasan P.S., Veeramani P. (2003). Fixed point for mappings satisfying cyclical contractive conditions. Fixed Point Theory.

[23] Lu N., He F., Du W. (2019). Fundamental questions and new counterexamples for b-metric spaces and Fatou property. Mathematics.

[24] Mohammadi B., Parvaneh V., Aydi H. (2019). On extended interpolative Ćirić-Reich-Rus type *F*-contractions and an application. J. Inequal. Appl..

[25] Reich S. (1971). Kannan's fixed point theorem. Boll. UMI.

[26] Reich S. (1972). Fixed points of contractive functions. Boll. UMI.

